# Characterising lower-body musculoskeletal morphology and whole-body composition of elite female and male Australian Football players

**DOI:** 10.1186/s13102-022-00561-8

**Published:** 2022-09-06

**Authors:** Callum J. McCaskie, Marc Sim, Robert U. Newton, Jarryd Heasman, Brent Rogalski, Nicolas H. Hart

**Affiliations:** 1 School of Medical and Health Sciences, Edith Cowan University, 270 Joondalup Drive, Joondalup, Perth, WA 6027 Australia; 2 West Coast Eagles Football Club, Perth, WA Australia; 3 Faculty of Health and Medical Sciences, The University of Western Australia, Perth, WA Australia; 4 Exercise Medicine Research Institute, Edith Cowan University, Perth, WA Australia; 5 School of Human Movement and Nutrition Sciences, University of Queensland, Brisbane, QLD Australia; 6 Caring Futures Institute, College of Nursing and Health Science, Flinders University Adelaide, Adelaide, SA Australia; 7 Institute for Health Research, University of Notre Dame Australia, Perth, WA Australia; 8 Centre for Healthcare Transformation, Queensland University of Technology, Brisbane, QLD Australia

**Keywords:** Muscle, Bone, Fat, Anthropometry, Density, Robustness

## Abstract

**Background:**

Physical demands and injury rates differ between elite female and male Australian Football (AF) players. To improve understanding of contributing physical factors to these differences, the purpose of this study was to investigate lower-body morphology and whole-body composition of elite footballers competing in the Australian Football League (AFL) and Australian Football League Women’s (AFLW).

**Methods:**

Lower-body morphology and whole-body composition of 23 AFL players and 23 AFLW players were assessed using peripheral Quantitative Computed Tomography and Dual-energy X-ray Absorptiometry at the beginning of pre-season. Differences between cohorts, with sub-analyses of kicking vs. support limbs, and experienced vs. inexperienced player status were assessed using two-sample independent t-tests. Magnitude of differences were assessed using Cohen’s *d* effect sizes.

**Results:**

AFL players had greater absolute (*p* < 0.001; ES = 3.28) and relative (*p* < 0.001; ES = 2.29) whole body lean soft-tissue mass, with less absolute (*p* = 0.004; ES = 0.91) and relative (*p* < 0.001; ES = 2.29) fat mass than AFLW players. For AFLW players, no significant differences existed between kicking and support limbs with few differences observed between experienced and inexperienced players.

**Conclusions:**

Greater emphasis on physical development in AFLW players may be required to enable increases in muscle mass and skeletal robustness, to ensure they can tolerate the loads of elite competition.

## Background

Australian Football (AF) is a field-based team sport played widely throughout Australia on large oval-shaped grounds [1]. At the elite level, the men’s game (Australian Football League; AFL) involves four 20-min quarters (excluding extra time) with 18 players on the field, and four interchange players [2], over 22 matches with a four-week finals series. Comparatively, the newly established elite women’s (AFLW) competition involves nine matches, with a three-week finals series (in 2021). While most of the rules are identical, several changes were made to the AFLW competition to reduce congestion, mitigate injury risk, and improve spectator experience [3]. These include shorter quarters (15-min vs. 20-min), less players on the field (16 vs. 18 players) and an extra interchange player (five vs. four).

Physical running demands of the AFL and AFLW are well documented [4], with the average elite male covering ~ 12 kms (km) per game at ~ 130 m per minute (m/min) [5] and the average elite female covering ~ 6 km per game at ~ 120 m/min [6]. Additionally, AFL players cover ~ 1800 m at speeds of or above 18 km/h [7, 8] with AFLW players covering only ~ 370 m at the same threshold [9]. While these absolute differences are somewhat influenced by shorter match durations in AFLW, it is likely that elite female players have different physical attributes to their male counterparts in response to different competitive demands. Exacerbating the likely difference in physical development between AFL and AFLW is their exposure to established developmental pathways, with a national talent pathway that includes physical development for males from the age of 14 [10], that is not presently available to females—a disparity that needs remedy. Furthermore, the physical and physiological differences between sexes are underpinned by variances in hormonal profile. Specifically, testosterone (which is naturally higher in males), is known to stimulate increased myogenesis and osteogenesis (muscle and bone mass) [11–13]. As activity requires a greater anaerobic contribution of energy, the physical and physiological disparities between males and females tend to widen, with greater lower-body strength, power and speed performance observed in elite German male soccer players [14]. Thus, understanding the differences in kinanthropometric profiles between male and female Australian footballers will provide practitioners with greater insight into how best prepare players for competition.

Exposure to football specific activities results in greater musculoskeletal indices in the support leg relative to the kicking leg over time [15] with differences observed between experienced (4 + years) and less-experienced players (1–3 years) in the AFL. However, the assessment of kinanthropometric characteristics of AFLW players and whether differences exist between limbs and experience levels is yet to be explored. In 2019, injury incidence (per 1000 player hours) was higher in the AFL than AFLW for all lower-body regions except anterior cruciate ligament (ACL) injuries (~ 700% higher in AFLW). Conversely, upper-body injuries such as shoulder, elbow, wrist, and hand had higher injury incidence in the AFLW competition. Collectively, due to differences between AFL and AFLW in injury epidemiology and physical demands, substantial differences in their kinanthropometric profile are likely to exist. The aims of this study were to examine lower-body morphology and whole-body composition of AFLW players compared to AFL players using Dual-energy X-ray Absorptiometry and peripheral Quantitative Computed Tomography. Second, we examined if differences exist between kicking and support limbs, as well as between inexperienced and experienced players in each cohort.

## Methods

A cross-sectional study design was used to collect kinanthropometric data of AFL and AFLW players (height, weight, body composition, lower-body morphology) at the beginning of their 2021 pre-seasons. Twenty-three AFL (mean ± SD; age = 21.4 ± 1.6 y; height = 186 ± 8 cm; body mass = 83.5 ± 8.3 kg; playing experience = 3.4 y) and twenty-three AFLW (mean ± SD; age = 25.8 ± 4.1 y; height = 169 ± 7 cm; body mass = 65.3 ± 6.7 kg; playing experience = 3.4 y) players from the same club participated in the study. Players were also divided into two groups for sub-analysis: (1) First or second season at the elite level (i.e., inexperienced players) and (2) Third season or more (i.e., experienced players). Any player who had recently undergone surgery, or a period of non-weight bearing activity or immobilisation within 6 months prior to data collection were not included. All AFLW and AFL players followed individualised off-season programs provided by club strength and conditioning specialists in the lead-up to their scans. Players were encouraged to log what was completed and not perform any additional exercise outside of their training program. Data was collected as part of normal club protocol which is part of players’ contractual arrangements. Ethics approval was provided by Edith Cowan University’s Human Research Ethics Committee (ID: 2020–01055).

Stature was recorded to the nearest 0.1 cm (cm) using a stadiometer (Model 217, Seca, Hamburg, Germany). Body mass was measured to the nearest 0.1 kg (kg) using electronic scales (Model 22089, Seca, Hamburg, Germany). Tibial length (to the nearest 0.1 cm) was measured using a retractable measuring tape (Model 4414; Tech-Med Service, NY, USA) from the bottom of the medial malleolus at the distal end of the tibia to the top of the tibial plateau at the knee joint [16].

Dual-energy X-ray Absorptiometry (DXA; Hologic Horizon-A, Danbury, CT, USA) was used to assess whole-body composition in accordance with scan procedures detailed previously [15]. Numerous players were too tall for the scanning region, thus the head was removed from analysis, resulting in whole body less head (WBLH) measures for each player to maintain consistency across the two cohorts. The same qualified operator analysed the scans by adjusting anatomical lines to separate the torso, arms, legs, pelvic and spine regions. Sub-regions were also created to separate the lower-limbs into thigh and shank segments [17]. WBLH fat mass (FM) and lean soft-tissue mass (LSTM) were obtained as well as FM and LSTM from each sub-region. LSTM refers to all fat-free soft-tissue mass and doesn’t include any hard-tissue (bone). The coefficient of variation (CV) for whole-body DXA scans in our facility, used by the same operator (CJM) for repeat scans on a subset of 30 individuals (males and females of varying ages and sizes) were as follows: total mass = 0.22%; LSTM = 0.41%; FM = 1.61% for the whole-body; LSTM = 0.95% and FM = 2.36% for the whole leg; LSTM = 1.02%; FM = 2.27% for the thigh; and LSTM = 1.73%, FM = 5.09% for the shank region.

Peripheral Quantitative Computed Tomography (pQCT; XCT-3000, Stratec Medizintechnik, Pforzeim, Germany) was used to assess musculoskeletal morphology (volumetric mass, volumetric density, and cross-sectional areas [CSA]) of the lower-legs (kicking and support limbs) separately using previously described scanning procedures [15]. Cross-sectional examinations at specific tibial sites (4%, 14%, 38% and 66% of tibial length—distal to proximal) were undertaken. Tibial mass, tibial area, and total volumetric bone mineral density (vBMD) were reported at individual tibial sites. Conversely, cortical thickness (CortTh), periosteal circumference (PeriC), endosteal circumference (EndoC) and polar stress–strain index (SSIPOL) are presented as averaged values across the 14% and 38% sites [15]. SSIPOL is an accurate indicator of long-bone structural properties and an estimator of bending-strength [18]. Relative fracture load (FL.Rel) was reported and represents the averaged absolute fracture load (N) of the X and Y-axes divided by player body mass (kg). Total tibial vBMD, total tibial mass (4%, 14% and 38%) and total cortical density (CortD; 14%, 38% and 66%) are presented as the average across three sites. The CV for repeat tibial pQCT scans of the left lower leg on a subset of four individuals [19] by the same operator (CJM) were as follows: Tibial mass = 0.62%; Tibial CSA = 0.80%; vBMD = 0.33%; CortTh = 0.78%; PeriC = 0.25%; EndoC = 0.40%; Muscle CSA = 0.44%; SSIPOL = 2.12%. A quality control cone phantom was also scanned every three days, and the CV for total attenuation for repeat scans was 0.14%. Tibial robustness was also acquired for the entire bone by calculating the averaged total CSA of the tibia across all four sites, and dividing it by tibial length to reflect the biological increase in width and length of bone (averaged total tibial CSA / tibial length) [20].

Data was prepared using Python (v3.7.6) in source-code editor Visual Studio code (v1.61.0) using numerous Python packages (Numpy, Pandas, Scipy, Seaborn and Matplotlib). All variables were assessed for normality using the Kolmogorov–Smirnov test. Variables which were not normally distributed were log-transformed before further analyses were conducted. Independent samples t-tests were utilised to compare the differences between (i) AFL and AFLW players, (ii) Inexperienced (< 3 years) and experienced (≥ 3 years) players within each cohort and (iii) Kicking and support leg within each cohort. Significance was set at ≤ 0.05. The magnitude of the difference for each analysis was assessed using Cohen’s *d* effect sizes (ES) [21]. Effect sizes were used as follows: 0.00–0.19 = trivial; 0.20–0.59 = small; 0.60–1.19 = moderate; 1.20–1.99 = large; ≥ 2.00 = very large [21]. Levene’s test was also used to assess the equality of variances.

## Results

**Fig. 1:**
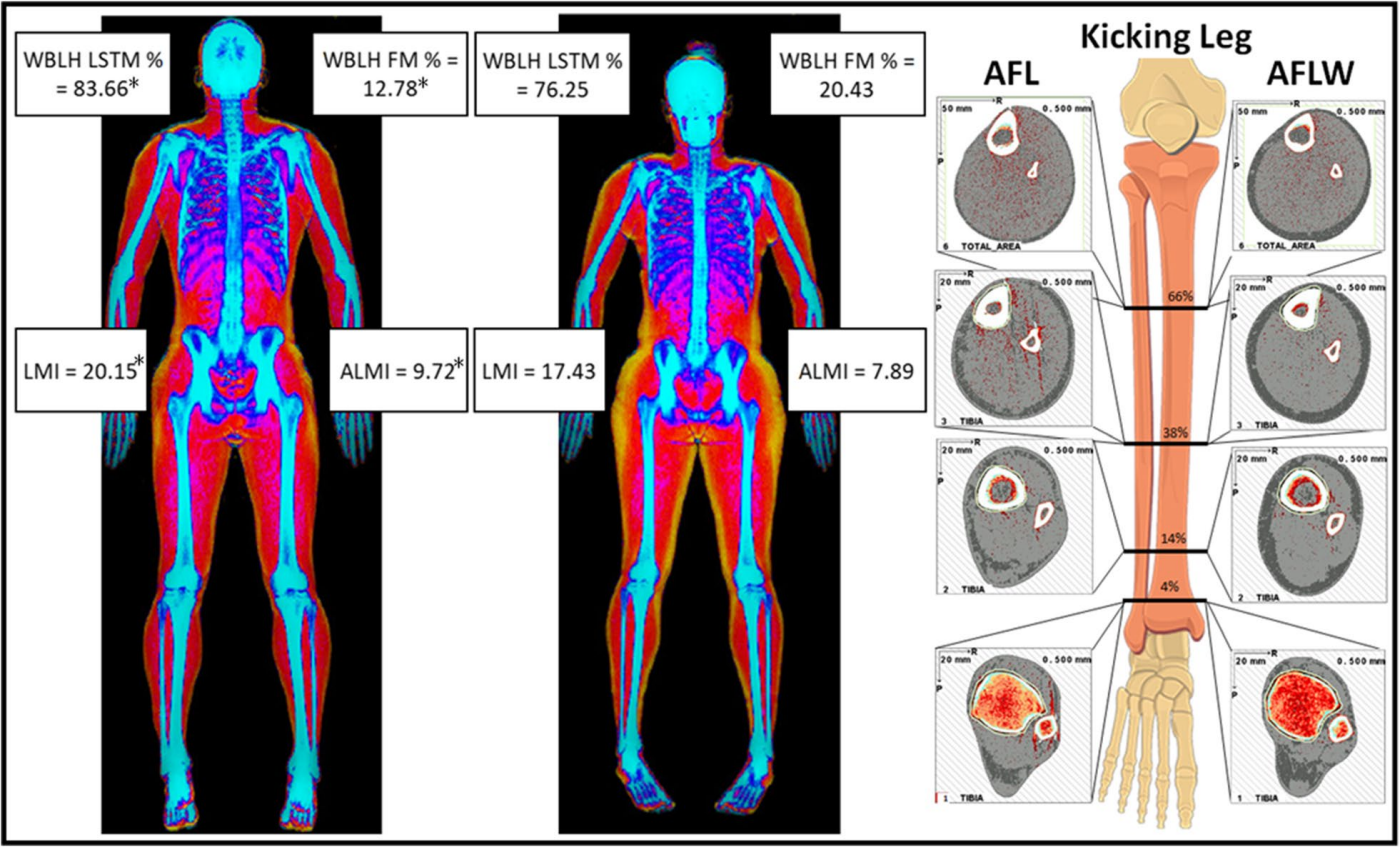
Body composition and lower-body musculoskeletal comparisons between AFL and AFLW players. ALMI = appendicular lean mass index; FM = fat mass; LMI = lean mass index; LSTM = lean soft-tissue mass; WBLH = whole body less head; *significantly (*p* < 0.05) different from AFLW

A visual representation of the body composition and lower-body musculoskeletal characteristics of an AFL and AFLW player are presented in Fig. 1.

AFL players had greater volumetric tibial mass and tibial CSA than AFLW players across all measured slices (4%, 14% and 38%) for the kicking leg and support leg (Table 1). However, total vBMD was only significantly greater in AFL players at the 4% slice. AFL players had superior CortTh, EndoC and PeriC for the kicking and support legs. For bone strength indices, SSIPOL and FL.Rel for kicking and support legs were significantly greater in AFL players than AFLW players. AFL players had 20% greater cross-sectional area of muscle (*p* < 0.001) and 53% less fat area (*p* < 0.001) at the 66% Tibial slice compared to AFLW players, with no difference in muscle density (Table 1).

**Table 1:** Kinanthropometric characteristics between kicking and support leg in AFL and AFLW players and the differences between AFL and AFLW

	AFL				AFLW				AFL v AFLW			
	Kicking	Support			Kicking	Support			Kicking	Support		
	mean ± SD	mean ± SD	*p*	*ES*	mean ± SD	mean ± SD	*p*	*ES*	*p*	*ES*	*p*	*ES*
*DXA*												
Total Leg FM (kg)	2.12 ± 0.43	2.15 ± 0.40	0.326	0.07	3.22 (0.96)	3.14 (1.14)	0.958	0.04	< 0.001*	1.46^d^	< 0.001*	1.52^d^
Total Leg LSTM (kg)	12.12 (1.84)	11.96 (1.53)	0.695	0.12	8.78 ± 0.92	8.68 ± 0.99	0.153	0.10	< 0.001*	3.08^e^	< 0.001*	3.19^e^
Total Leg FM%	13.8 ± 2.31	14.1 ± 2.09	0.102	0.14	25.1 ± 4.99	25.2 ± 4.69	0.707	0.02	< 0.001*	2.91^e^	< 0.001*	3.06^e^
Thigh FM (kg)	1.52 ± 0.31	1.50 ± 0.36	0.681	0.06	2.34 (0.62)	2.29 (0.69)	0.985	0.02	< 0.001*	1.58^d^	< 0.001*	1.62^d^
Thigh LSTM (kg)	8.64 (1.20)	8.84 (1.20)	0.686	0.12	6.10 (1.14)	6.04 (1.07)	0.651	0.13	< 0.001*	3.07^e^	< 0.001*	3.16^e^
Thigh FM%	14.0 ± 2.40	14.4 ± 2.15	0.033*	0.18^a^	26.1 ± 4.80	26.3 ± 4.65	0.366	0.04	< 0.001*	3.18^e^	< 0.001*	3.30^e^
Shank FM (kg)	0.48 ± 0.17	0.54 ± 0.26	0.215	0.26	0.73 ± 0.28	0.70 ± 0.24	0.127	0.09	< 0.001*	1.08^c^	< 0.001*	1.05^c^
Shank LSTM (kg)	2.91 ± 0.41	2.93 ± 0.41	0.387	0.05	2.03 (0.40)	2.06 (0.37)	0.919	0.03	< 0.001*	2.31^e^	< 0.001*	2.31^e^
Shank FM%	12.9 ± 3.26	13.2 ± 2.62	0.399	0.10	23.8 ± 7.08	23.3 ± 6.19	0.195	0.08	< 0.001*	1.98^d^	< 0.001*	2.12^e^
*pQCT*												
4% Bone Mass (g)	5.33 ± 0.67	5.38 ± 0.65	0.439	0.08	3.83 ± 0.46	3.90 ± 0.47	0.228	0.14	< 0.001*	2.60^e^	< 0.001*	2.60^e^
14% Bone Mass (g)	3.59 ± 0.39	3.68 ± 0.38	0.017*	0.21^b^	2.77 (0.56)	2.82 (0.53)	0.794	0.07	< 0.001*	1.92^d^	< 0.001*	2.14^e^
38% Bone Mass (g)	5.16 ± 0.54	5.31 ± 0.52	0.010*	0.28^b^	3.95 (0.48)	3.91 (0.57)	0.995	0.01	< 0.001*	2.10^e^	< 0.001*	2.38^e^
Total Tibial Mass (g)	4.69 ± 0.49	4.79 ± 0.49	0.044*	0.19^a^	3.50 (0.57)	3.55 (0.60)	0.794	0.07	< 0.001*	2.45^e^	< 0.001*	2.59^e^
4% Bone Area (mm^2^)	1363 (197)	1400 (208)	0.552	0.18	1108 ± 112	1109 ± 117	0.922	0.01	< 0.001*	1.68^d^	< 0.001*	1.79^d^
14% Bone Area (mm^2^)	596.3 ± 83.9	620.3 ± 84.2	0.001*	0.29^b^	492.3 ± 55.7	494.2 ± 48.3	0.759	0.04	< 0.001*	1.46^d^	< 0.001*	1.84^d^
38% Bone Area (mm^2^)	567.8 ± 63.9	581.2 ± 63.7	0.023*	0.21^b^	430.3 (36.8)	421.3 (68.8)	0.951	0.02	< 0.001*	2.15^e^	< 0.001*	2.28^e^
66% Bone Area (mm^2^)	953 (151)	971 (126)	0.838	0.09	747.5 ± 93.0	754.0 ± 96.6	0.327	0.07	< 0.001*	1.88^d^	< 0.001*	2.11^e^
4% vBMD (mg/cm^3^)	388.8 ± 47.1	382.7 ± 47.3	0.177	0.13	346.7 ± 31.9	352.7 ± 34.3	0.116	0.18	0.001*	1.05^c^	0.018*	0.73^c^
14% vBMD (mg/cm^3^)	608.5 ± 61.2	597.3 ± 54.3	0.016*	0.19^a^	587.0 ± 49.6	589.0 ± 50.8	0.690	0.04	0.198	0.39	0.595	0.16
38% vBMD (mg/cm^3^)	910.8 ± 35.8	915.1 ± 36.4	0.142	0.12	918.9 ± 30.5	917.1 ± 30.7	0.658	0.06	0.415	0.24	0.844	0.06
Total vBMD (mg/cm^3^)	636.0 ± 38.1	631.7 ± 37.9	0.122	0.11	617.5 ± 29.9	619.6 ± 29.2	0.490	0.07	0.074	0.54	0.231	0.36
Total CortD (mg/cm^3^)	1118 (29.4)	1116 (33.3)	0.841	0.06	1141 ± 16.3	1140 ± 16.2	0.946	0.01	< 0.001*	1.62^d^	< 0.001*	1.62^d^
Total CortTh (mm)	4.96 ± 0.39	5.01 ± 0.37	0.249	0.12	4.20 (0.62)	4.21 (0.57)	0.960	0.02	< 0.001*	1.99^d^	< 0.001*	2.24^e^
Total PeriC (mm)	84.4 (4.1)	86.2 (4.2)	0.338	0.29	75.7 (5.06)	75.7 (4.0)	0.902	0.03	< 0.001*	1.98^d^	< 0.001*	2.29^e^
Total EndoC (mm)	54.15 ± 5.02	55.2 ± 4.81	0.008*	0.22^b^	50.0 ± 3.37	50.18 ± 3.14	0.602	0.05	< 0.001*	0.97^c^	< 0.001*	1.25^d^
SSIPOL (mm^3^)	2461 (334)	2660 (312)	0.166	0.42	1737 (337)	1757 (334)	0.862	0.04	< 0.001*	1.97^d^	< 0.001*	2.30^e^
FL.Rel (N/kg)	68.7 (7.0)	70.0 (8.6)	0.392	0.25	60.2 (12.4)	57.3 (6.1)	0.545	0.16	0.007*	0.84^c^	< 0.001*	1.19^c^
Tibial robustness	2.02 (0.28)	2.07 (0.31)	0.496	0.18	1.87 ± 0.13	1.88 ± 0.13	0.586	0.08	< 0.001*	1.18^c^	< 0.001*	1.38^d^
66% Muscle Area (mm^2^)	8444 ± 1212	8403 ± 1219	0.475	0.03	6893 ± 799	6872 ± 919	0.773	0.03	< 0.001*	1.51^d^	< 0.001*	1.42^d^
66% MuscleD (mg/cm^3^)	79.7 (2.0)	79.8 (1.6)	0.600	0.20	79.4 ± 1.3	79.2 ± 1.6	0.685	0.14	0.770	0.13	0.532	0.21
66% Fat Area (mm^2^)	1282 ± 355	1264 ± 390	0.527	0.05	2203 ± 650	2251 ± 664	0.133	0.07	< 0.001*	1.76^d^	< 0.001*	1.81^d^

Data is presented as mean ± SD or Median (IQR) for non-normally distributed variables

*CortD* cortical density; *CortTh* cortical thickness; *DXA* dual-energy x-ray absorptiometry; *EndoC* endosteal circumference; *FL.Rel* relative fracture load; *FM* fat mass; *LSTM* lean soft-tissue mass; *MuscleD* muscle density; *PeriC* periosteal circumference; *pQCT* peripheral Quantitative Computed Tomography; *SSIPOL* polar stress–strain index; *vBMD* volumetric bone mineral density

^*^denotes significance (*p* < 0.05)

^a^Trivial effect size (< 0.2)

^b^Small effect size (0.2–0.59)

^c^Moderate effect size (0.6–1.19)

^d^Large effect size (1.2–1.99)

^e^Very large effect size (≥ 2.00)

In the AFL (male) cohort, lower-body morphological differences were mostly evident at the 14% Tibial slice between kicking and support legs, with tibial mass and CSA being key differentiators (Table 1). In contrast, no differences were observed between kicking and support legs for any body composition or morphological variable in the AFLW cohort (Table 1).

Inexperienced AFL players had significantly less WBLH LSTM (*p* = 0.002; ES = 1.57), with greater whole leg FM% (*p* = 0.048; ES = 1.00) and thigh FM% (*p* = 0.037; ES = 0.99). For morphological variables, no differences existed between groups for total vBMD, FL.Rel and CortTh (Table 2). Tibial mass at the 14% site was 10% greater in the experienced group, with Tibial CSA greater in the experienced group by 13.8%, 10.7% and 15.7% at the 14%, 38% and 66% tibial sites respectively. Interestingly, experienced players had 15.8% greater cross-sectional area of the gastrocnemius on the support leg (*p* = 0.022) (Table 3), but the difference was not significant for the kicking leg (*p* = 0.069) (Table 2). Conversely, the only differences seen between experienced and inexperienced AFLW players were for tibial area at the 66% site (10.5% greater in experienced; *p* = 0.048) and FL.Rel (10.6% greater in experienced; *p* = 0.04).

**Table 2:** Body composition and musculoskeletal morphology of the kicking leg between inexperienced and experienced players for AFL and AFLW players

	AFL				AFLW			
	Inexperienced (n = 6)	Experienced (n = 17)			Inexperienced (n = 10)	Experienced (n = 13)		
	*Mean* ± *SD*	*Mean* ± *SD*	*p*	*ES*	*Mean* ± *SD*	*Mean* ± *SD*	*p*	*ES*
*General*								
Age (y)	19.7 ± 1.5	21.9 ± 1.3	0.002*	1.57^d^	23.8 ± 4.5	27.3 ± 3.2	0.040*	0.90^c^
Height (cm)	183 ± 9.6	188 ± 6.9	0.227	0.54	168 ± 6.8	171 ± 6.3	0.258	0.49
Body mass (kg)	78 ± 7.5	86 ± 7.8	0.055	0.98	66 ± 7.5	65 ± 6.2	0.809	0.10
Playing Year (y)	1.5 (1.0)	4.0 (1.0)	0.001*	3.95^e^	2.0 (1.0)	5.0 (0.0)	< 0.001*	5.64^e^
*DXA*								
WBLH LSTM (kg)	61.2 ± 6.90	68.7 ± 6.46	0.025*	1.12^c^	46.4 ± 3.82	47.7 ± 4.86	0.518	0.30
WBLH FM (kg)	10.5 ± 1.75	10.1 ± 1.99	0.082	0.87	14.0 ± 4.27	11.88 ± 2.71	0.106	0.70
WBLH LSTM%	82.4 ± 2.13	84.1 ± 2.04	0.103	0.82	74.8 ± 4.37	77.4 ± 3.34	0.124	0.67
WBLH FM%	14.1 ± 2.01	12.3 ± 2.06	0.082	0.87	22.0 ± 4.60	19.2 ± 3.54	0.106	0.70
LMI (kg/m^2^)	19.2 ± 1.39	20.5 ± 0.99	0.019*	1.10^c^	17.6 ± 1.26	17.3 ± 0.89	0.453	0.31
Appendicular LMI (kg/m^2^)	9.12 (1.35)	9.99 (0.78)	0.058	0.88	7.86 ± 0.70	7.91 ± 0.55	0.866	0.07
Total Leg LSTM (kg)	11.69 (1.98)	12.30 (2.45)	0.069	0.90	8.61 ± 0.69	8.91 ± 1.07	0.451	0.33
Total Leg FM%	15.4 ± 2.13	13.2 ± 2.15	0.048*	1.00^c^	26.4 ± 5.40	24.1 ± 4.62	0.276	0.47
Thigh LSTM (kg)	8.38 ± 0.82	9.23 ± 1.05	0.087	0.90	6.18 ± 0.51	6.38 ± 0.80	0.482	0.30
Thigh FM%	15.7 ± 2.64	13.4 ± 2.06	0.037*	0.99^c^	27.3 ± 5.30	25.1 ± 4.36	0.285	0.45
Shank LSTM (kg)	2.70 ± 0.35	2.99 ± 0.41	0.147	0.76	2.00 ± 0.24	2.15 ± 0.31	0.197	0.54
Shank FM%	14.1 ± 3.16	12.5 ± 3.29	0.319	0.49	25.3 ± 7.75	22.7 ± 6.59	0.388	0.37
*pQCT*								
Tibial length (mm)	413 ± 22.3	426 ± 21.6	0.234	0.577	367 ± 13.4	377 ± 22.0	0.206	0.57
4% Bone Mass (g)	4.95 ± 0.58	5.46 ± 0.66	0.114	0.81	3.87 ± 0.43	3.80 ± 0.50	0.731	0.15
14% Bone Mass (g)	3.33 ± 0.25	3.69 ± 0.38	0.044*	1.12^c^	2.80 ± 0.27	2.95 ± 0.41	0.340	0.42
38% Bone Mass (g)	4.81 ± 0.57	5.29 ± 0.48	0.056	0.92	3.94 ± 0.29	4.16 ± 0.62	0.298	0.47
Total Tibial Mass (g)	4.36 ± 0.43	4.81 ± 0.47	0.051	1.00	3.54 ± 0.32	3.64 ± 0.47	0.563	0.25
4% Bone Area (mm^2^)	1294 ± 112	1414 ± 223	0.225	0.68	1096 ± 96.3	1116 ± 125	0.677	0.18
14% Bone Area (mm^2^)	537 ± 52.5	617 ± 83.8	0.041*	1.15^c^	479 ± 51.1	502 ± 59.2	0.354	0.40
38% Bone Area (mm^2^)	524 ± 60.7	583 ± 59.1	0.048*	0.99^c^	431 (31.8)	429 (77.6)	0.286	0.50
66% Bone Area (mm^2^)	891 (98.3)	982 (126)	0.025*	1.21^d^	703 ± 72.6	782 ± 94.7	0.040*	0.94^c^
4% vBMD (mg/cm^3^)	385 ± 59.3	390 ± 44.1	0.833	0.09	354 ± 38.6	341 ± 25.8	0.325	0.41
14% vBMD (mg/cm^3^)	623 ± 54.7	603 ± 64.2	0.520	0.32	586 ± 48.0	588 ± 52.8	0.930	0.04
38% vBMD (mg/cm^3^)	918 ± 41.5	908 ± 34.6	0.588	0.25	920 ± 34.9	918 ± 28.0	0.919	0.04
Total vBMD (mg/cm^3^)	642 ± 39.7	634 ± 38.6	0.671	0.20	620 ± 34.0	616 ± 27.7	0.739	0.14
Total CortD (mg/cm^3^)	1122 (25.0)	1116 (33.5)	0.605	0.58	1140 ± 13.9	1141 ± 18.4	0.808	0.11

Data is presented as mean ± SD or Median (IQR) for non-normally distributed variables

*CortD* cortical density; *CortTh* cortical thickness; *DXA* dual-energy x-ray absorptiometry; *EndoC* endosteal circumference; *FL.Rel* relative fracture load; *FM* fat mass; *LMI* lean mass index; *LSTM* lean soft-tissue mass; *PeriC* periosteal circumference; *pQCT* peripheral Quantitative Computed Tomography; *SSIPOL* polar stress–strain index; *vBMD* volumetric bone mineral density; *WBLH* whole body less head

*denotes significance (*p* < 0.05)

^a^Trivial effect size (< 0.2)

^b^Small effect size (0.2–0.59)

^c^Moderate effect size (0.6–1.19)

^d^Large effect size (1.2–1.99)

^e^Very large effect size (≥ 2.00)

**Table 3:** Body composition and musculoskeletal morphological characteristics of the support leg between inexperienced and experienced players for AFL and AFLW players

	AFL				AFLW			
	Inexperienced (n = 6)	Experienced (n = 17)			Inexperienced (n = 10)	Experienced (n = 13)		
	*Mean* ± *SD*	*Mean* ± *SD*	*p*	*ES*	*Mean* ± *SD*	*Mean* ± *SD*	*p*	*ES*
*General*								
Age (y)	19.7 ± 1.5	21.9 ± 1.3	0.002*	1.57^d^	23.8 ± 4.5	27.3 ± 3.2	0.040*	0.90^c^
Height (cm)	183 ± 9.6	188 ± 6.9	0.227	0.54	168 ± 6.8	171 ± 6.3	0.258	0.49
Body mass (kg)	78 ± 7.5	86 ± 7.8	0.055	0.98	66 ± 7.5	65 ± 6.2	0.809	0.10
Playing Year (y)	1.5 (1.0)	4.0 (1.0)	0.001*	3.95^e^	2.0 (1.0)	5.0 (0.0)	< 0.001*	5.64^e^
*DXA*								
WBLH LSTM (kg)	61.2 ± 6.90	68.7 ± 6.46	0.025*	1.12^c^	46.4 ± 3.82	47.7 ± 4.86	0.518	0.30
WBLH FM (kg)	10.5 ± 1.75	10.1 ± 1.99	0.082	0.87	14.0 ± 4.27	11.88 ± 2.71	0.106	0.70
WBLH LSTM%	82.4 ± 2.13	84.1 ± 2.04	0.103	0.82	74.8 ± 4.37	77.4 ± 3.34	0.124	0.67
WBLH FM%	14.1 ± 2.01	12.3 ± 2.06	0.082	0.87	22.0 ± 4.60	19.2 ± 3.54	0.106	0.70
LMI (kg/m^2^)	19.2 ± 1.39	20.5 ± 0.99	0.019*	1.10^c^	17.6 ± 1.26	17.3 ± 0.89	0.453	0.31
Appendicular LMI (kg/m^2^)	9.12 (1.35)	9.99 (0.78)	0.058	0.88	7.86 ± 0.70	7.91 ± 0.55	0.866	0.07
Total Leg LSTM (kg)	11.4 ± 1.04	12.8 ± 1.26	0.030*	1.21^d^	8.48 ± 0.85	8.83 ± 1.09	0.410	0.36
Total Leg FM%	15.8 ± 1.51	13.5 ± 1.96	0.016*	1.32^d^	26.8 ± 4.93	23.9 ± 4.25	0.146	0.63
Thigh LSTM (kg)	8.21 ± 0.84	9.13 ± 0.96	0.051	1.02^c^	6.08 ± 0.60	6.28 ± 0.74	0.495	0.30
Thigh FM%	15.9 ± 2.25	13.8 ± 1.91	0.036*	1.02^c^	27.9 ± 5.03	25.1 ± 4.11	0.158	0.61
Shank LSTM (kg)	2.68 ± 0.34	3.01 ± 0.40	0.082	0.89	2.01 ± 0.27	2.17 ± 0.32	0.212	0.54
Shank FM%	14.8 ± 2.1	12.7 ± 2.61	0.087	0.90	25.1 ± 6.49	21.9 ± 5.83	0.230	0.52
*pQCT*								
Tibial length (mm)	413.2 ± 22.3	425.8 ± 21.55	0.234	0.58	367 ± 13.4	377.3 ± 22.0	0.206	0.57
4% Bone Mass (g)	4.92 ± 0.54	5.54 ± 0.62	0.042*	1.06^c^	3.86 ± 0.48	3.93 ± 0.48	0.761	0.13
14% Bone Mass (g)	3.43 ± 0.36	3.76 ± 0.36	0.060	0.94	2.73 (0.55)	2.82 (0.52)	0.415	0.34
38% Bone Mass (g)	5.00 ± 0.53	5.42 ± 0.48	0.090	0.82	3.97 (0.52)	3.91 (0.68)	0.547	0.30
Total Tibial Mass (g)	4.45 ± 0.45	4.91 ± 0.45	0.044*	1.02^c^	3.56 ± 0.36	3.67 ± 0.46	0.536	0.27
4% Bone Area (mm^2^)	1288 ± 163	1468 ± 217	0.080	0.94	1088 ± 112	1124 ± 123	0.480	0.30
14% Bone Area (mm^2^)	557 ± 71.4	643 ± 78.2	0.028*	1.14^c^	484 ± 48.7	502 ± 48.4	0.384	0.37
38% Bone Area (mm^2^)	545 ± 58.5	594 ± 62.03	0.106	0.81	422 (72.3)	421 (66.6)	0.439	0.36
66% Bone Area (mm^2^)	909 ± 88.3	994 ± 110	0.104	0.85	711 (99.6)	746 (93.4)	0.055	0.83
4% vBMD (mg/cm^3^)	386 ± 56.7	382 ± 45.4	0.858	0.08	356 ± 43.2	350 ± 27.2	0.667	0.18
14% vBMD (mg/cm^3^)	618 ± 43.7	590 ± 56.92	0.284	0.56	588 ± 51.5	590 ± 52.4	0.955	0.02
38% vBMD (mg/cm^3^)	918 ± 31.1	914 ± 38.9	0.804	0.13	921 ± 30.9	914 ± 31.4	0.573	0.24
Total vBMD (mg/cm^3^)	641 ± 32.8	629 ± 40.0	0.509	0.34	622 ± 32.9	618 ± 27.2	0.739	0.14
Total CortD (mg/cm^3^)	1118 ± 17.0	1109 ± 19.8	0.356	0.47	1138 ± 15.2	1142 ± 17.2	0.521	0.28
Total CortTh (mm)	4.96 ± 0.42	5.03 ± 0.37	0.685	0.19	4.30 (0.54)	4.01 (0.70)	0.990	0.02
Total PeriC (mm)	83.1 ± 4.5	88 ± 4.35	0.028*	1.11^c^	75.1 (4.85)	75.7 (3.84)	0.378	0.39
Total EndoC (mm)	51.9 ± 4.28	56.39 ± 4.53	0.048*	1.01^c^	49.4 ± 2.93	50.8 ± 3.26	0.277	0.47
SSIPOL (mm^3^)	2387 ± 370	2735 ± 377	0.064	0.93	1715 (352)	1781 (306)	0.380	0.40
FL.Rel (N/kg)	70.8 ± 9.58	71.6 ± 6.82	0.834	0.09	56.8 (3.85)	58.9 (14.2)	0.185	0.57
Tibial Robustness	2.00 ± 0.16	2.17 ± 0.23	0.096	0.86	1.85 ± 0.12	1.90 ± 0.13	0.391	0.40
66% Muscle Area (mm^2^)	7448 ± 1143	8740 ± 1083	0.022*	1.16^c^	6881 ± 981	6865 ± 909	0.969	0.02

Data is presented as mean ± SD or Median (IQR) for non-normally distributed variables

*CortD* cortical density; *CortTh* cortical thickness; *DXA* dual-energy x-ray absorptiometry; *EndoC* endosteal circumference; *FL.Rel* relative fracture load; *FM* fat mass; *LMI* lean mass index; *LSTM* lean soft-tissue mass; *PeriC* periosteal circumference; *pQCT* peripheral Quantitative Computed Tomography; *SSIPOL* polar stress–strain index; *vBMD* volumetric bone mineral density; *WBLH* whole body less head

*denotes significance (*p* < 0.05)

^a^Trivial effect size (< 0.2)

^b^Small effect size (0.2–0.59)

^c^Moderate effect size (0.6–1.19)

^d^Large effect size (1.2–1.99)

^e^Very large effect size (≥ 2.00)

## Discussion

This is the first study to compare lower-body musculoskeletal morphology and whole-body composition of elite female and male Australian footballers (AFLW and AFL respectively). Apart from total vBMD, all other bone characteristics were significantly greater in AFL players compared to AFLW across the kicking and support limbs. AFL players also had more absolute WBLH and segmental LSTM and significantly less FM.

Similar results were seen in a study comparing kinanthropometric characteristics of male and female collegiate soccer players, with males displaying significantly greater LSTM of the total body and legs with significantly less relative whole body FM [22]. Furthermore, most of the musculoskeletal morphology differences between soccer players were observed at the 4% site of the tibia (vBMD, bone mass) with no differences observed at the 66% site. In the present study, vBMD could only differentiate between AFL and AFLW players at the 4% site, with no significant differences at any other sites, for either limb. This result was also reflected in another study which found vBMD could not differentiate between AF players of varied experience or between limbs despite significant differences seen in bone area, bone mass and bone strength indices [15], highlighting bone density as a poor indicator of bone strength when used in isolation [23, 24]. Significant differences between AFL and AFLW were seen at all tibial sites for other bone indices including tibial mass, tibial CSA and CortTh. It has previously been reported that sex differences in musculoskeletal characteristics exist due to many factors including hormonal processes and different sensitivity to mechanical loading [25, 26]. Hart and colleagues [23] outlined that muscle plays a pivotal role in bone strength, providing mechanical protection and repairing skeletal tissue. Therefore, the finding that AFL players have superior bone characteristics than AFLW players is not surprising, given that AFL players have greater quantities of whole body and segmental LSTM.

While absolute LSTM is associated with superior athletic performance [27], inter-limb asymmetry may be problematic. Even 3% asymmetry in LSTM between kicking and support legs equated to ~ 8% difference in strength and could explain differences in kicking accuracy in sub-elite AF players [28]. Large inter-limb strength asymmetries have also been associated with a heightened injury risk [29, 30]. Kinanthropometric differences between kicking and support limbs in elite male Australian footballers have been identified previously [15, 16], with the support leg displaying greater tibial mass, and greater trabecular, cortical and total tibial CSA than the kicking leg. Similar results were seen in the current study, confirming prior results, with tibial mass at the 14% and 38% sites and tibial CSA at the 14% and 38% sites greater (*p* < 0.05) in the support leg in the AFL cohort. Furthermore, the support leg had greater EndoC. Such findings may be related to the asymmetrical and unipedal loading patterns over time, with the support leg exposed to frequent high-grade axial load impacts (i.e. during kicking, and single-leg jumping as examples), with relatively fewer incidences in the kicking leg [15]. Interestingly, no musculoskeletal morphology characteristics were significantly different between kicking and support limbs for AFLW players, which may reflect their limited exposure to AF at developmental and elite levels. Indeed, while AFL and AFLW cohorts had ‘similar experience’ at the elite level, a large proportion of AFLW players came from other sporting codes (e.g., netball, soccer, rugby 7’s) prior to competing in the AFLW. Thus, they have not had the same longitudinal exposure to the game as AFL players who have been exposed to junior leagues and the national talent and local development pathways.

Exposure to the physical and mechanical AFL training and match demands will likely have a substantial influence on players’ kinanthropometric profile, with the soft- and hard-tissues of the body becoming more resilient as they adapt to this load over time. Accordingly, the current study examined the differences in lower-body morphology and whole-body composition between inexperienced and experienced players. Many confirmatory parallels were noted between this work and previous research [15], with experienced AFL players observed to have significantly greater relative amounts of LSTM and less relative WBLH, total leg and thigh FM. Previous research has established that younger, inexperienced players have a higher risk of injury [31], and one factor might be that these players do not have the musculoskeletal development or resilience to tolerate the loads required at AFL level in their early years. Experienced players had greater tibial bone characteristics, particularly at the 14% and 38% sites bilaterally, including greater tibial mass and tibial area, similar to previous work [16]. The tibia is the most common site for stress fracture in athletic populations [32], with most of these stress responses occurring at the distal third of the tibia [33, 34], in similar areas to the 14% and 38% slices. Thus, it could be suggested that longitudinal exposure to football-specific loads also increases the tolerance of hard-tissue in these stress-prone regions. Alternatively, the AFLW season is only nine games long with formalised training lasting five months of the year. Subsequently, minimal differences were seen in the AFLW cohort between inexperienced and experienced players and between limbs. This may suggest that the AFLW season is not long enough to induce musculoskeletal adaptations or to differentiate between players of varying experience levels. Longitudinal loading has been shown to increase musculoskeletal asymmetry between limbs [15]. However, many AFLW players have not had the same developmental exposure as their AFL counterparts, which may indicate they are physically under-prepared for the sport. Thus, a greater emphasis on their physical development may need to be a priority for AFLW practitioners.

Strengths of this study include the use of elite female and elite male athletes at comparable timepoints in their respective seasons. Furthermore, DXA and pQCT were utilised with musculoskeletal morphological characteristics examined at the 4%, 14%, 38% and 66% slices of the tibiae, allowing comparison to many different athletic populations as these slices are used abundantly in the literature. However, this study was not without limitations. Players were only recruited from one professional Australian football club (with an AFLW and AFL team in each competition), which may not provide an accurate representation for all players across the entire league. However, it should be noted that our AFL results in this study aligned strongly with those published previously from a rival team [16]. Given that AFLW is still in its infancy, and satisfactory development and talent pathways are currently being developed for female players, future research should examine multiple AFLW clubs to confirm the findings of this study. Additionally, a comprehensive examination into the players physical activity and nutritional history was not undertaken and may provide greater insight into the lower-body morphology and whole-body composition characteristics they exhibited. For AFLW players, information surrounding menstrual history and contraceptive use would also provide more context regarding their bone morphological traits.

## Conclusions

Large differences in lower-body morphology and whole-body composition exist between AFL and AFLW players. Whilst most skeletal traits associated with athletic performance appear superior in the AFL cohort, no differences existed for vBMD, highlighting that BMD has limited utility in evaluating bone strength and should be used in conjunction with other bone measures, such as bone mass and structure. AFL players also had greater asymmetry between kicking and support limbs with significantly greater skeletal qualities in their support leg. Significantly greater bone traits were also observed in experienced players versus inexperienced, exemplifying the influence of longitudinal loading. Conversely, no differences between limbs or between experience groups were observed for the AFLW cohort. This may highlight the need for a longer AFLW pre-season and the establishment of development pathways to ensure maximum physical development in these female players to prepare them for the demands of AFLW match play.

## Data Availability

The datasets used and/or analysed during the current study, including all individual de-identified data are not publicly available due to the agreement with the football club.

## Abbreviations

ACL: Anterior cruciate ligament
AF: Australian football
AFL: Australian football league
AFLW: Australian football league women’s
ALMI: Appendicular lean mass index
CortD: Cortical density
CortTh: Cortical thickness
CSA: Cross-sectional area
CV: Coefficient of variation
DXA: Dual-energy x-ray absorptiometry
EndoC: Endosteal circumference
ES: Effect size
FL.Rel: Relative Fracture Load
FM: Fat mass
LMI: Lean mass index
LSTM: Lean soft-tissue mass
PeriC: Periosteal circumference
pQCT: Peripheral quantitative computed tomography
SSIPOL: Polar stress–strain index
vBMD: Volumetric bone mineral density
WBLH: Whole body less head
