# Correction: Characterising lower-body musculoskeletal morphology and whole-body composition of elite female and male Australian Football players

**DOI:** 10.1186/s13102-022-00564-5

**Published:** 2022-09-22

**Authors:** Callum J. McCaskie, Marc Sim, Robert U. Newton, Jarryd Heasman, Brent Rogalski, Nicolas H. Hart

**Affiliations:** 1 School of Medical and Health Sciences, Edith Cowan University, 270 Joondalup Drive, 6027 Joondalup, Perth, WA Australia; 2 West Coast Eagles Football Club, Perth, WA Australia; 3 Faculty of Health and Medical Sciences, The University of Western Australia, Perth, WA Australia; 4 Exercise Medicine Research Institute, Edith Cowan University, Perth, WA Australia; 5 School of Human Movement and Nutrition Sciences, University of Queensland, Brisbane, QLD Australia; 6 Caring Futures Institute, College of Nursing and Health Science, Flinders University Adelaide, Adelaide, SA Australia; 7 Institute for Health Research, University of Notre Dame Australia, Perth, WA Australia; 8 Centre for Healthcare Transformation, Queensland University of Technology, Brisbane, QLD Australia

## Correction: BMC Sports Sci Med Rehabil 14, 168 (2022) https://doi.org/10.1186/s13102-022-00561-8

Following publication of the original article [1], the authors identified a typesetting error. Table 2 was not published in full and was missing the last 7 rows. The complete Table 2 is supplied in this correction article.

The original article [1] has been corrected.

**Table 2:** Body composition and musculoskeletal morphology of the kicking leg between inexperienced and experienced players for AFL and AFLW players

	AFL				AFLW			
	**Inexperienced (n = 6)**	**Experienced** ** (n = 17)**			**Inexperienced (n = 10)**	**Experienced** ** (n = 13)**		
	***Mean*** ± ***SD***	***Mean*** ± ***SD***	***p***	***ES***	***Mean*** ± ***SD***	***Mean*** ± ***SD***	***p***	***ES***
**General**								
Age (y)	19.7 ± 1.5	21.9 ± 1.3	0.002*	1.57^d^	23.8 ± 4.5	27.3 ± 3.2	0.040*	0.90^c^
Height (cm)	183 ± 9.6	188 ± 6.9	0.227	0.54	168 ± 6.8	171 ± 6.3	0.258	0.49
Body mass (kg)	78 ± 7.5	86 ± 7.8	0.055	0.98	66 ± 7.5	65 ± 6.2	0.809	0.10
Playing Year (y)	1.5 (1.0)	4.0 (1.0)	0.001*	3.95^e^	2.0 (1.0)	5.0 (0.0)	< 0.001*	5.64^e^
**DXA**								
WBLH LSTM (kg)	61.2 ± 6.90	68.7 ± 6.46	0.025*	1.12^c^	46.4 ± 3.82	47.7 ± 4.86	0.518	0.30
WBLH FM (kg)	10.5 ± 1.75	10.1 ± 1.99	0.082	0.87	14.0 ± 4.27	11.88 ± 2.71	0.106	0.70
WBLH LSTM%	82.4 ± 2.13	84.1 ± 2.04	0.103	0.82	74.8 ± 4.37	77.4 ± 3.34	0.124	0.67
WBLH FM%	14.1 ± 2.01	12.3 ± 2.06	0.082	0.87	22.0 ± 4.60	19.2 ± 3.54	0.106	0.70
LMI (kg/m^2^)	19.2 ± 1.39	20.5 ± 0.99	0.019*	1.10^c^	17.6 ± 1.26	17.3 ± 0.89	0.453	0.31
Appendicular LMI (kg/m^2^)	9.12 (1.35)	9.99 (0.78)	0.058	0.88	7.86 ± 0.70	7.91 ± 0.55	0.866	0.07
Total Leg LSTM (kg)	11.69 (1.98)	12.30 (2.45)	0.069	0.90	8.61 ± 0.69	8.91 ± 1.07	0.451	0.33
Total Leg FM%	15.4 ± 2.13	13.2 ± 2.15	0.048*	1.00^c^	26.4 ± 5.40	24.1 ± 4.62	0.276	0.47
Thigh LSTM (kg)	8.38 ± 0.82	9.23 ± 1.05	0.087	0.90	6.18 ± 0.51	6.38 ± 0.80	0.482	0.30
Thigh FM%	15.7 ± 2.64	13.4 ± 2.06	0.037*	0.99^c^	27.3 ± 5.30	25.1 ± 4.36	0.285	0.45
Shank LSTM (kg)	2.70 ± 0.35	2.99 ± 0.41	0.147	0.76	2.00 ± 0.24	2.15 ± 0.31	0.197	0.54
Shank FM%	14.1 ± 3.16	12.5 ± 3.29	0.319	0.49	25.3 ± 7.75	22.7 ± 6.59	0.388	0.37
**pQCT**								
Tibial length (mm)	413 ± 22.3	426 ± 21.6	0.234	0.577	367 ± 13.4	377 ± 22.0	0.206	0.57
4% Bone Mass (g)	4.95 ± 0.58	5.46 ± 0.66	0.114	0.81	3.87 ± 0.43	3.80 ± 0.50	0.731	0.15
14% Bone Mass (g)	3.33 ± 0.25	3.69 ± 0.38	0.044*	1.12^c^	2.80 ± 0.27	2.95 ± 0.41	0.340	0.42
38% Bone Mass (g)	4.81 ± 0.57	5.29 ± 0.48	0.056	0.92	3.94 ± 0.29	4.16 ± 0.62	0.298	0.47
Total Tibial Mass (g)	4.36 ± 0.43	4.81 ± 0.47	0.051	1.00	3.54 ± 0.32	3.64 ± 0.47	0.563	0.25
4% Bone Area (mm^2^)	1294 ± 112	1414 ± 223	0.225	0.68	1096 ± 96.3	1116 ± 125	0.677	0.18
14% Bone Area (mm^2^)	537 ± 52.5	617 ± 83.8	0.041*	1.15^c^	479 ± 51.1	502 ± 59.2	0.354	0.40
38% Bone Area (mm^2^)	524 ± 60.7	583 ± 59.1	0.048*	0.99^c^	431 (31.8)	429 (77.6)	0.286	0.50
66% Bone Area (mm^2^)	891 (98.3)	982 (126)	0.025*	1.21^d^	703 ± 72.6	782 ± 94.7	0.040*	0.94^c^
4% vBMD (mg/cm^3^)	385 ± 59.3	390 ± 44.1	0.833	0.09	354 ± 38.6	341 ± 25.8	0.325	0.41
14% vBMD (mg/cm^3^)	623 ± 54.7	603 ± 64.2	0.520	0.32	586 ± 48.0	588 ± 52.8	0.930	0.04
38% vBMD (mg/cm^3^)	918 ± 41.5	908 ± 34.6	0.588	0.25	920 ± 34.9	918 ± 28.0	0.919	0.04
Total vBMD (mg/cm^3^)	642 ± 39.7	634 ± 38.6	0.671	0.20	620 ± 34.0	616 ± 27.7	0.739	0.14
Total CortD (mg/cm^3^)	1122 (25.0)	1116 (33.5)	0.605	0.58	1140 ± 13.9	1141 ± 18.4	0.808	0.11
Total CortTh (mm)	4.89 ± 0.45	4.99 ± 0.38	0.617	0.23	4.17 ± 0.32	4.25 ± 0.40	0.615	0.22
Total PeriC (mm)	81.5 ± 3.34	86.7 ± 4.59	0.020*	1.28^d^	74.8 (4.55)	76.1 (5.75)	0.293	0.47
Total EndoC (mm)	50.8 ± 4.0	55.3 ± 4.90	0.054	1.02	49.2 ± 3.08	50.6 ± 3.58	0.342	0.41
SSIPOL (mm^3^)	2226 ± 257	2576 ± 345	0.034*	1.15^c^	1725 (262)	1737 (376)	0.275	0.51
FL.Rel (N/kg)	68.6 ± 9.28	69.9 ± 6.44	0.726	0.15	58.6 ± 4.49	65.7 ± 10.9	0.048*	0.85^c^
Tibial Robustness	1.96 ± 0.10	2.13 ± 0.24	0.022*	0.92^c^	1.84 ± 0.12	1.89 ± 0.14	0.408	0.38
66% Muscle Area (mm^2^)	7675 ± 1318	8715 ± 1084	0.069	0.86	6998 ± 888	6813 ± 751	0.595	0.22

Data is presented as mean ± SD or Median (IQR) for non-normally distributed variables

*CortD* cortical density; *CortTh* cortical thickness; *DXA* dual-energy x-ray absorptiometry; *EndoC* endosteal circumference; *FL.Rel* relative fracture load; *FM* fat mass; *LMI* lean mass index; *LSTM* lean soft-tissue mass; *PeriC* periosteal circumference; *pQCT* peripheral Quantitative Computed Tomography; *SSIPOL* polar stress–strain index; *vBMD* volumetric bone mineral density; *WBLH* whole body less head

*denotes significance (*p* < 0.05)

^a^Trivial effect size (< 0.2)

^b^Small effect size (0.2–0.59)

^c^Moderate effect size (0.6–1.19)

^d^Large effect size (1.2–1.99)

^e^Very large effect size (≥ 2.00)
